# Discovery of ferroptosis-related genes in renal ischemia reperfusion and evaluate the potential impact on kidney transplantation

**DOI:** 10.3389/fimmu.2024.1394477

**Published:** 2024-09-06

**Authors:** Yao Zhou, Yuwei Yang, Bo Wang, Wan Chen, Yanlin Wei, Ruihua Wu, LingZhang Meng, Liwen Lyu

**Affiliations:** ^1^ Department of Emergency, Guangxi Academy of Medical Sciences & People’s Hospital of Guangxi Zhuang Autonomous Region, Nanning, China; ^2^ Ruikang Hospital Affiliated to Guangxi University of Chinese Medicine, Nanning, China; ^3^ Institute of Cardiovascular Sciences, Guangxi Academy of Medical Sciences, & The People’s Hospital of Guangxi Zhuang Autonomous Region, Nanning, China

**Keywords:** acute kidney injury, ferroptosis, bioinformatics, proteomics, kidney transplantation

## Abstract

**Background:**

Renal ischemia reperfusion injury (IRI) is one of the pivotal event of acute kidney injury (AKI), and they are unavoidable in the process of kidney transplantation, which eventually leads to the loss of renal allograft. Ferroptosis is a newly identified programmed cell death. Recent studies have suggested that ferroptosis may participate in the pathophysiological process of renal IRI. Therefore, we aimed to determine biomarkers associated with ferroptosis during renal IRI and their impact on renal allografts.

**Methods:**

We conducted a comprehensive bioinformatics analysis and established an IRI-AKI animal model to illustrate the critical role of ferroptosis-related hub genes (FRHGs) in IRI-AKI and their potential impact on kidney transplantation.

**Results:**

In this study, we identified 60 ferroptosis-related genes (FRGs) in renal IRI based on the GSE148420 dataset and FerrDb database. And then we performed functional annotation analysis using Gene Ontology (GO) and Kyoto Encyclopedia of Genes and Genomes (KEGG) pathway enrichment. Protein-protein interaction (PPI) network was constructed by online tool String. EZH2, CDKN1A, PPARA, EGR1, ATF3, and CD44 were identited as six ferroptosis-related hubgenes (FRHGs) using four methods, including MMC, Degree, DMNC, and EPC. FRHGs expression level were verified by the validation sets GSE58438 and GSE126805. Protein expression level of FRHGs verified by Proteomics and Western blot. Cibersort was utilized to analyze immune cell infiltration during renal IRI as well as the correlation between FRHGs and immune cells. The GSE21374 dataset was used for renal allografts survival analysis. Finally, We induced the IRI-AKI animal model and illustrated the importance of FRGHs CD44 in ferroptosis and the accumulation of macrophages.

**Conclusion:**

We identified 6 FRHGs. We found that FRHGs not only exhibited significant correlation with immune cells but also directly influenced the survival of transplanted kidneys in the human population. Among six FRHGs, only CD44 was overexpressed at both the gene and protein levels. Anti-CD44 exerts a protective effect by inhibiting ferroptosis and the accumulation of M1 macrophages during renal IRI. This study provided new insights into the pathogenesis of renal IRI and provided new evidence for its treatment.

## Introduction

Renal IRI, which can cause acute kidney injury (AKI), is a common event after kidney transplantation, increases the risk of delayed graft function (DGF), rejects the donor kidney, and leads to a long-term risk of allograft loss. Existing research has shown that metabolic imbalance and excessive generation of reactive oxygen species (ROS) during the hypoxia process, as well as inflammatory reactions during the re-oxygenation process are important pathological and physiological processes in renal IRI (1).

Ferroptosis is a novel form of programmed cell death characterized by iron-dependent phospholipid peroxidation (2). It is caused by an imbalance between oxidants and antioxidants, which is driven by the abnormal expression and activity of various oxidoreductases (3). Oxidative stress is a crucial factor in renal IRI. Recent studies have suggested that ferroptosis may play an important role in IRI (4, 5). For example, legumain has been shown to promote renal tubule ferroptosis in IRI-AKI, and this mechanism may be related to chaperone-mediated autophagy of GPX4 (6). However, further research is needed to explore how ferroptosis is involved in the development and progression of renal IRI.

In this study, we download GSE148420, GSE58438, GSE126805 and GSE21374 datasets from the Gene Expression Omnibus (GEO) database. And then we performed a systematic bioinformatic analysis based on differentially expressed ferroptosis-related genes (DEFRGs), so as to identify FRHGs involved in the renal IRI process in the kidneys and analyze their associations with immune infiltration and renal allograft survival. Finally, We performed proteomic analysis and animal experiment to further validate the expression of FRHGs and the ferroptosis-related pathways enriched during renal IRI.

## Materials and methods

### Data collection and acquisition of ferroptosis-related genes

**Figure 1:**
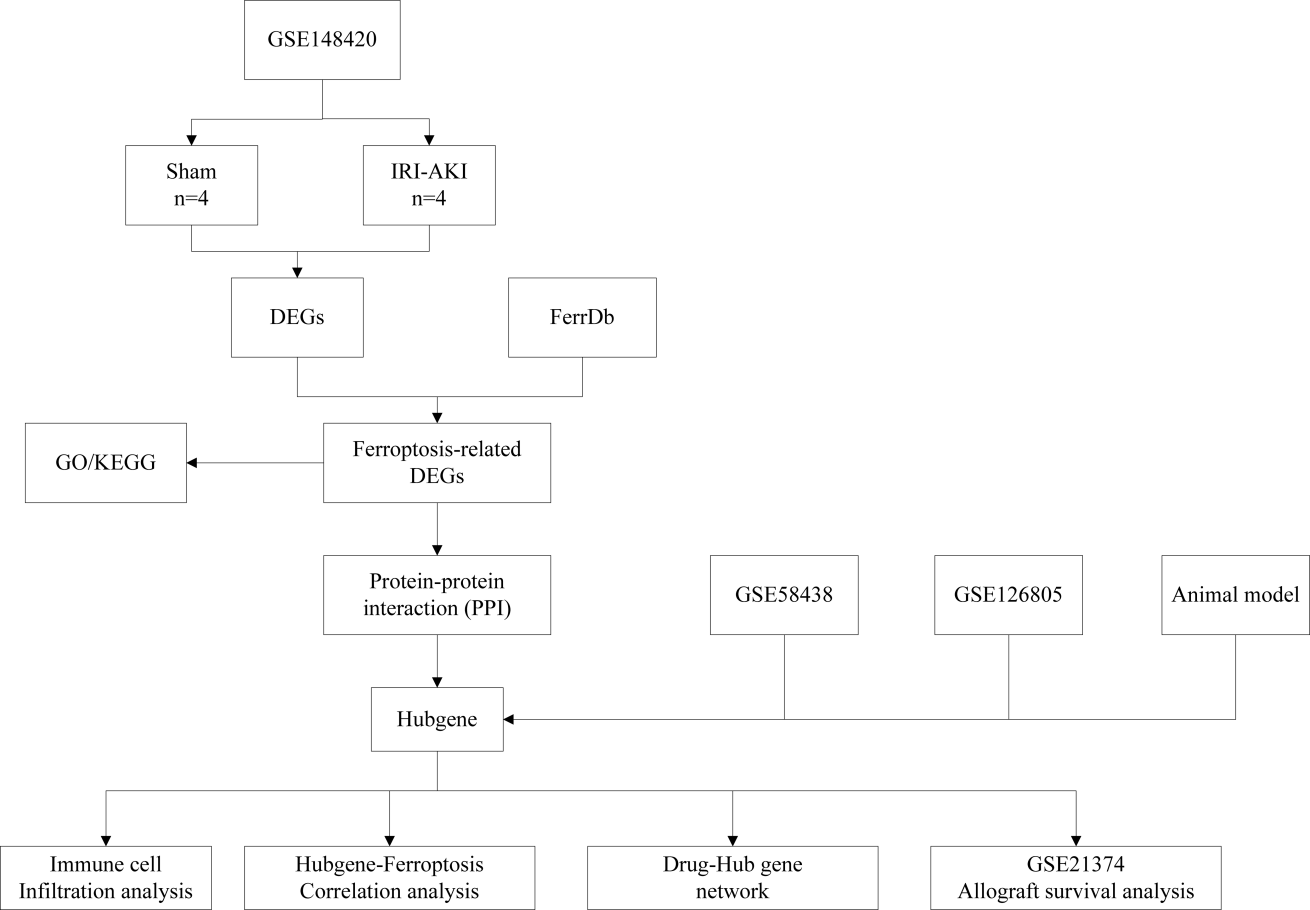
Flowchart of this study.

The RNA expression data, which included 4 IRI groups and 4 Sham groups, were collected from GEO (http://www.ncbi.nlm.nih.gov/geo/) database with series numbers GSE148420. The GSE21374, GSE58438 and GSE126805 datasets were also obtained from the GEO database. The dataset GSE58438 contains 19 samples from 4 experimental groups, including 5 Sham group samples, five IRI 3h samples, four IRI 24h samples, and five IRI 120h samples. The dataset GSE126805 includes samples from 42 kidney transplant recipients at 4 different time points, namely before transplant, after transplant, 3 months after transplant, and 1 year after transplant. The dataset GSE21374 includes gene expression data and graft survival information from renal transplant patients, comprising 51 cases of allograft loss and 231 cases of allograft survival. These datasets cover various stages of ischemia-reperfusion injury, but unfortunately, they come from different species. Ferroptosis-related genes(FRGs), including drive, suppress, or mark gene, were obtained from the FerrDb (http://www.zhounan.org/ferrdb) database. A flowchart of this study is shown in **Figure 1**.

### Establishment of animal models

Eighteen SPF-grade Balb/c mice were selected, aged 6-8 weeks, weighing 20-25g (purchased from Hunan SJA Laboratory Animal Co., Ltd), and were randomly divided into Sham group, IR-AKI group and IR+Anti-CD44 group. The IR+Anti-CD44 group was injected with CD44 monoclonal antibody (100ug) 30 minutes before ischemia procedure. The surgical incision was made approximately 0.5 cm to the left or right of the midline on the back of the mouse to expose the kidney. The right kidney was removed from all groups, the Sham group exposed the left kidney without clamping, and the IR group exposed the left kidney and rapidly clamped the left renal pedicle with an atraumatic microvascular clamp. Successful clamping was indicated by a rapid change in kidney color from dark red to dark purple. After 45 minutes of ischemia, releasing the clamp demonstrated a change in kidney color from purple-black back to red, indicating successful reperfusion. The reperfusion time was 24 hours. This study has been approved by the Animal Ethics Committee of Guangxi University of Chinese Medicine (Approval No.: DW20220529-216).

### HE and PAS staining

Hematoxylin and eosin (HE) staining and PAS staining was used to detect pathological damage in the kidney tissue. All steps were performed according to manufacturer’s instructions. The injury scoring was recorded with blind assessment method, with the following scores: 0, normal; 1, mild; 2, moderate; 3, severe.

### Identification of differentially expressed ferroptosis-related genes

Differentially-expressed genes (DEGs) between IRI-AKI and Sham group were acquired by the “Limma” package in R software. The absolute value of Log2Fold > 1.0 and adj.P<0.05 were used as significance indicators. DEGs were visualized by volcanic maps using the “ggplot2” package in R software. The top 50 DEGs were shown by heat map based on the “pheatmap” package in R software. The intersection of DEGs and FRGs obtained in the FerrDb Database is defined as differentially expressed ferroptosis-related genes (DEFRGs). The number of DEFRGs was shown in a Venn diagram using the online tool “jvenn” (https://jvenn.toulouse.inrae.fr) (7).

### Functional annotation and pathway enrichment of DEFGRs

To reveal the underlying biological functions and underlying mechanisms of DEFRGs, we used the Sangerbox (http://sangerbox.com/) online analysis tool for gene ontology (GO) and Kyoto Encyclopedia of Gene and Genes (KEGG) enrichment analysis of DEFRGs. GO enrichment analysis includes biological processes (BPs), cellular components (CCs) and molecular functions (MFs); adj. P<0.05 is the threshold for screening the main enrichment functions and pathways of DEFRGs.

### Construction of protein–protein interaction network of DEFRGs

To further explore the Protein–Protein interactions(PPI) among DEFRGs, we constructed a PPI network of DEFGRs using the online tool “String” (http://www.string-db.org/) (Version 11.5)database. We set 0.4 (medium confidence) as minimum interaction score. We identified key genes in the PPI network of DEFGRs using four algorithms from the Cytohubba plug-in. The four algorithms are Degree, Density of Maximum Neighborhood Component(DMNC), Edge Percolated component (EPC) and Maximum neighborhood component (MNC). Top 10 key genes were defined as hubgenes. Then the Hubgenes obtained from the four algorithms was intersected with each other, and the intersection genes were defined as the most valuable ferroptosis-related Hub genes (FRHGs) for renal IRI.

### Validation of FRHGs expression of IRI-AKI

To further validate the expression level of FRHGs in GSE148420, the external datasets GSE58438, GSE126805 were used as external validation sets. we performed a statistical analysis on the expression of the FRHGs between each group in the external validation set, and P<0.05 was considered statistically significant. Secondly, Proteomics and Western blot is used to validate the expression levels of key proteins. Proteomic sequencing is performed by Novogene Co. Ltd. Bioinformatics analysis was done with R software. An appropriate amount of liver tissue is taken and added to 500 μL of protein lysis buffer, then ground using a fully automatic cryogenic grinder. The resulting mixture is then centrifuged at low temperature and high speed (4°C,12,000 r/min) for 10 minutes, and the supernatant is collected to obtain the extracted tissue protein solution. After quantifying the protein using the BCA method, the protein is mixed with protein loading buffer at a 4:1 ratio and boiled at 100°C for 10 minutes. 60 μg of the sample is loaded per lane, and the protein extracts are separated by polyacrylamide gel electrophoresis (PAGE) in the electrophoresis solution. The proteins are then transferred to a PVDF membrane using the “wet transfer method.” The membrane is blocked with 5% skim milk at room temperature for 1 hour, followed by respective incubation with primary and secondary antibodies, and then visualized and analyzed using Imaging J.

### Correlation analysis between FRHGs and immune cell infiltration

R package “Cibersort” was used to calculate the proportion of 22 different immune cells type in GSE148420 datasets (8). Spearman’s correlation analysis between infiltrating immune cells and FRHGs was calculated using “Corrplot” in R software. Correlation analysis result was shown in the dot plot.

### Renal graft survival analysis

We further investigated the effects of FRHGs on long-term allograft survival through the utilization of the GSE21374 dataset. The Sangerbox online tool was used to plot survival curves.

### Potential drug identification of the FRHGs

We use the drug gene interactions database (DGIdb, www.dgidb.org) to predict drugs and molecular compounds that may interact with FRHGs. The Cytoscape software is used to visualize the drug-gene interaction network.

### Assessment of kidney function

Renal function was evaluated through the measurement of serum creatinine(Scr) and blood urea nitrogen(BUN)levels, conducted in accordance with the manufacturer’s protocol (Jiancheng, Nanjing, China).

### Measurement of MDA and GSH

For the Malondialdehyde (MDA) assay, an appropriate quantity of tissue sample was weighed, thoroughly lysed, and centrifuged to yield 100 μL of supernatant. The assay was subsequently performed following the provided kit instructions (Solarbio, China), with absorbance recorded at 532 nm and 600 nm using a microplate reader. The assessment of Glutathione (GSH) was conducted in accordance with the guidance of the GSH detection kit (Solarbio, China), measuring absorbance at 412 nm. All procedures were conducted on ice to preserve sample integrity.

### Immunohistochemistry and immunofluorescence detection

Tissue samples were fixed in 4% paraformaldehyde and rehydrated through a graded series of ethanol solutions. To block non-specific binding, sections were incubated at room temperature for 30 minutes in 10% goat serum. Subsequently, the sections were exposed to primary antibodies—mouse monoclonal antibodies against CD11b (EPR1344, Abcam), CD86 (EPR28721, Abcam), and GPX4 (67763-1-Ig, Proteintech)-and incubated overnight at 4°C. Following this, the samples were incubated with a secondary antibody for 30 minutes at room temperature. For immunohistochemistry (IHC), HRP-conjugated secondary antibodies were employed, while fluorescently labeled secondary antibodies were utilized for immunofluorescence (IF). Microscopic examination of the samples was conducted to assess staining.

### TUNEL staining

Apoptotic cells were identified using a TUNEL Apoptosis Assay Kit (K1123, APExBIO), following the manufacturer’s instructions. The average number of TUNEL-positive cells was quantified from five randomly selected fields under a fluorescence microscope (BX51, OLYMPUS, Japan) and expressed as a percentage of the total cell nuclei.

### Transmission electron microscopy

Small pieces of renal cortex were excised and fixed in 2.5% glutaraldehyde. Following fixation, the samples were dehydrated and embedded. Ultra-thin sections were stained and subsequently examined using a transmission electron microscope (HITACHI HT7800, Japan).

### Statistical analysis

All bioinformatics analyses were performed using R software; Statistical analyses of experimental data were processed by Prism software 9.0 (GraphPad software, La Jolla, CA). **** represents P<0.0001, *** represents P<0.001, ** represents P<0.01, * represents P<0.05.

## Results

### Identification of DEFRGs in IRI-AKI

**Figure 2:**
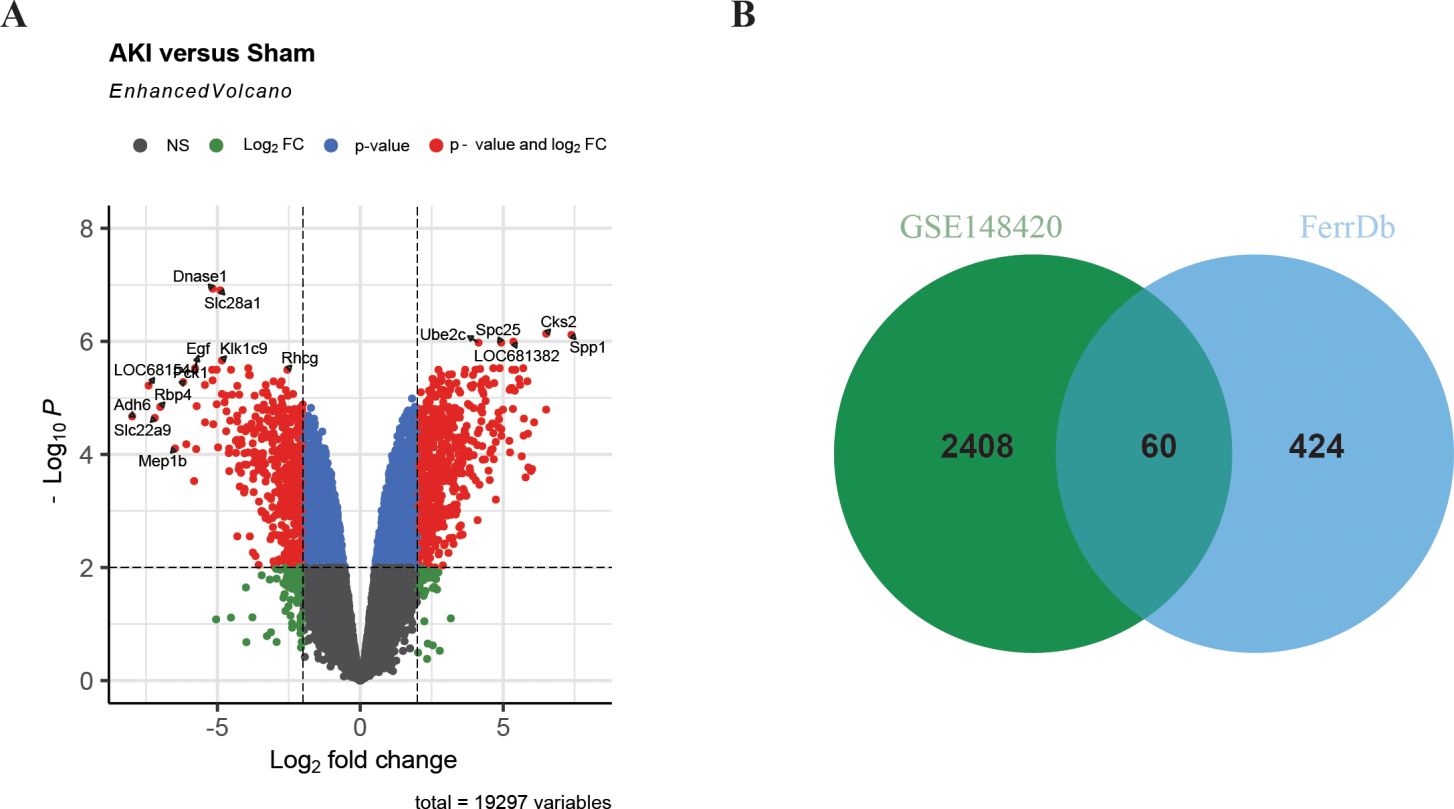
Identification of DEFRGs. **(A)** Volcano plot of differentially expressed genes in renal IRI. **(B)** Venn plot of Ferroptosis-related genes and DEGs in renal IRI.

In order to study the differentially expressed FRGs in IRI-AKI, 484 FRGs were extracted from FerrDb. FerrDb is a database of markers and diseases related to ferroptosis, which includes ferroptosis markers, drivers, and suppressors. Differential expression analysis of the GSE148420 dataset showed that 2,468 genes were significantly differentially expressed in IRI-AKI group compared to sham group, with a threshold of |log2FC|≥ 1 and adj.P < 0.05 (**Figure 2A**). After taking the intersection of DEGs and FRGs, a total of 60 differentially expressed FRGs were defined as DEFRGs (**Figure 2B**), which were presented in a Venn diagram.

### Functional enrichment analysis of DEFRGs

**Figure 3:**
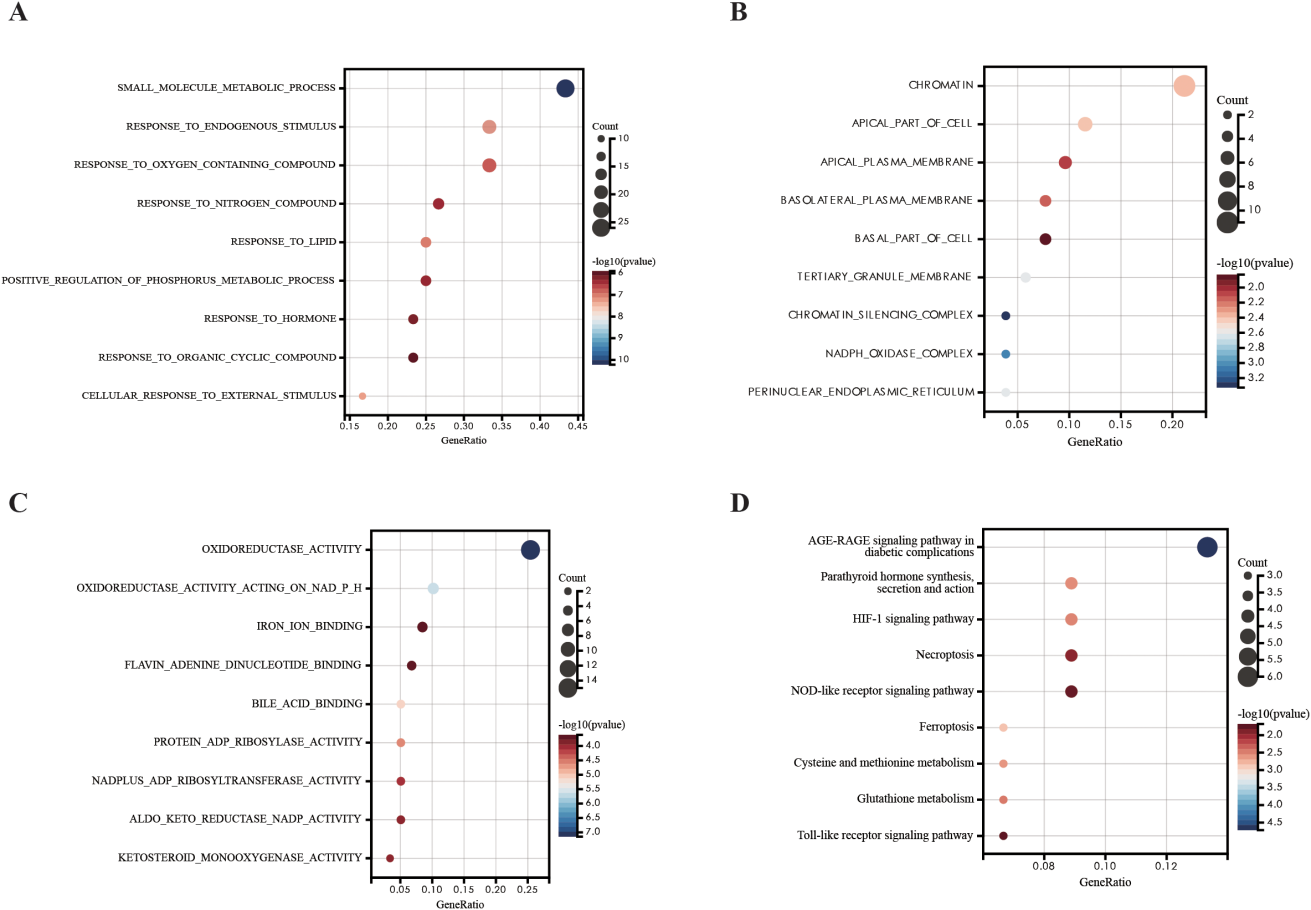
GO and KEGG pathway analysis of DEFRGs. **(A)** GO-BP analysis of DEFRGs. **(B)** GO-MF analysis of DEFRGs. **(C)** GO-CC analysis of DEFRGs. **(D)** KEGG analysis of DEFRGs.

We investigated the potential biological function and pathways of DEFRGs based on GO and KEGG pathway analysis. The results were presented in a dot plot. GO-BP analysis revealed that the DEFRGs were mainly enriched in “small molecule metabolic process”,”response to lipid”,” response to endogenous stimulus”[biological process, (BP)] (**Figure 3A**). GO-MF analysis revealed that the DEFRGs were mainly enriched in “oxidoreductase activity”, “oxidoreductase activity acting on NADP(H)”,”iron ion binding” (**Figure 3B**). In the GO-CC, DEFRGs were mostly enriched in the “chromatin”,” apical part of cell”, “apical plasma membrane”,” basolateral plasma membrane”,” basal part of cell”,” tertiary granule membrane” “chromatin silencing complex” “NADPH oxidase complex “, “perinuclear endoplasmic reticulum” [cellular component, (CC)] (**Figure 3C**). According to KEGG enrichment analysis, these DEFRGs were mainly enriched in the “HIF-1 signaling pathway”, “ Toll-like receptor signaling pathway”, “NOD-like receptor signaling pathway” and “Ferroptosis” (**Figure 3D**).

### Protein–protein interaction network construction and visualization

**Figure 4:**
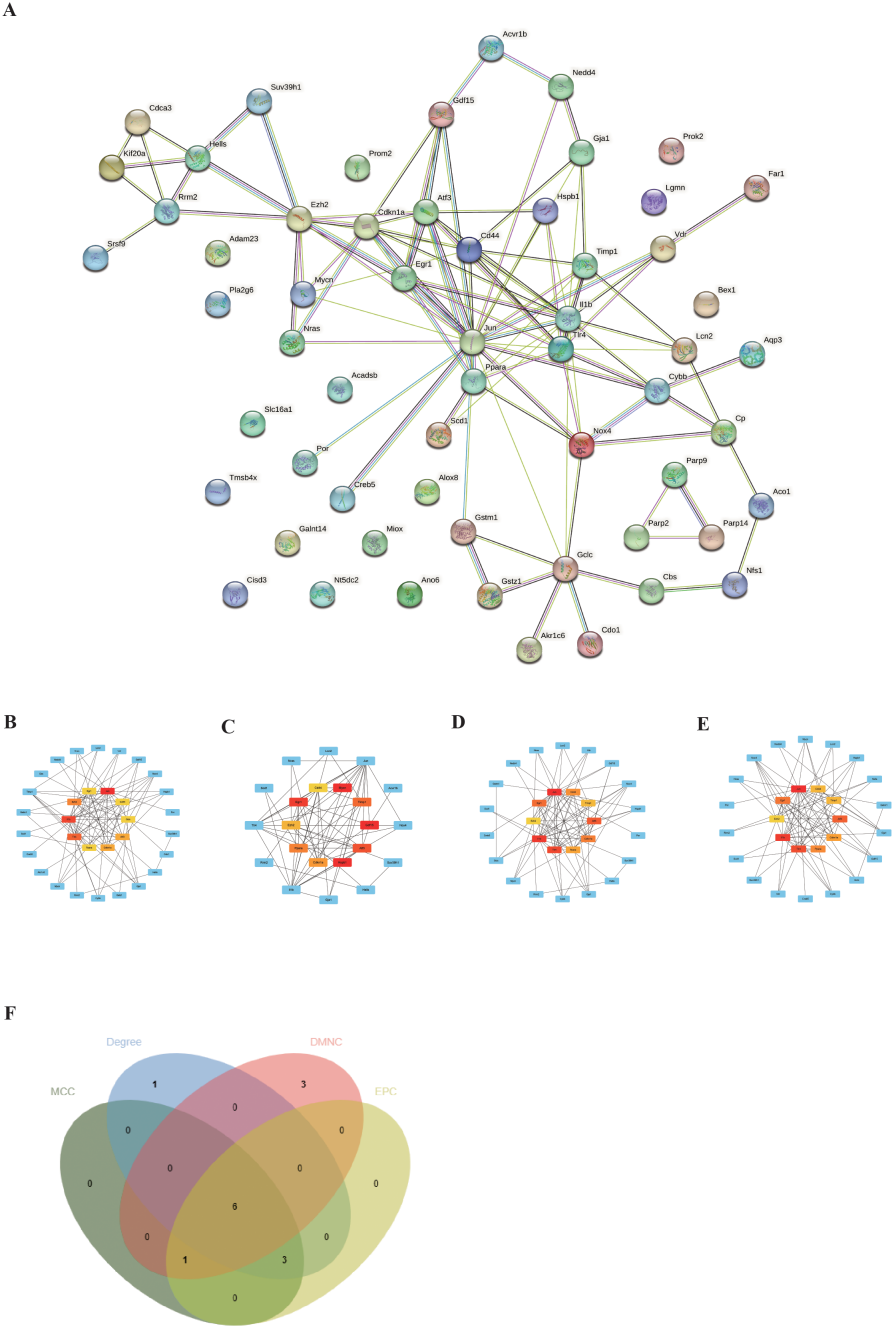
Identification of FRHGs. **(A)** PPI network of DEFRGs. **(B)** Top 10 Hubgenes identified by the Degree algorithm. **(C)** Top 10 Hubgenes identified by the DMNC algorithm. **(D)** Top 10 Hubgenes identified by the MNC algorithm. **(E)** Top 10 Hubgenes identified by the EPC algorithm. **(F)** Intersection of Hubgenes identified by all four algorithms.

To explore the interactions between each DEFRG, all DEFRGs were submitted to the STING database to construct a PPI network. The DEFRGs PPI network included 59 nodes and 106 edges (**Figure 4A**). Subsequently, the PPI data was imported into Cytoscape, and four algorithms, namely Degree (**Figure 4B**), DMNC (**Figure 4C**), MNC (**Figure 4D**), and EPC (**Figure 4E**), were applied in Cytohubba to analyze hubgenes. The top 10 hubgenes obtained from the four algorithms were intersected, resulting in six ferroptosis-related hubgenes (FRHGs), including ATF3, EGR1, PPARA, CDKN1A, CD44, and EZH2 (**Figure 4F**).

### Validation of FRHGs and allograft survival analysis

**Figure 5:**
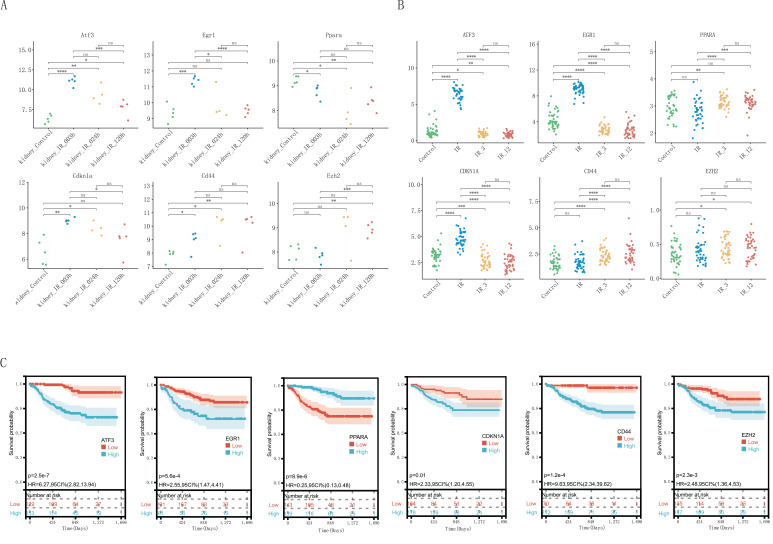
Validation and graft survival analysis of FRHGs. **(A)** FRHGs expression level in GSE58438. **(B)** FRHGs expression level in GSE126805. **(C)** The relationship between FRHGs and renal allograft survival. **** represents P<0.0001, *** represents P<0.001, ** represents P<0.01, * represents P<0.05, and ns indicates no statistical significance or no statistical difference.

Through the above analysis process, we identified as EZH2, CDKN1A, PPARA, EGR1, ATF3, and CD44 as FRHGs. The datasets GSE58438 and GSE126805 were used to further confirm the expression levels of the FRHGs at different time points. In GSE58438, ATF3, EGR1, CDKN1A, and CD44 were significantly upregulated as early as 3 hours after renal IRI, where ATF3 and CD44 continued to increase for up to 120 hours. On the other hand, PPARA showed a decreasing trend at 3 hours after renal IRI, which continued to decrease for up to 120 hours. The increase in EZH2 expression was observed at around 120 hours (**Figure 5A**). In GSE126805, a similar trend was observed for ATF3, EGR1, and CDKN1A, whereas CD44 and EZH2 continued to increase even up to 1 year after renal IRI (**Figure 5B**). Interestingly, PPARA showed an increasing trend at 3 months after IR, which continued to increase for up to 1 year. To further understand the role of FRHGs in IRI-AKI, GSE21374 was utilized to analyze the relationship between FRHGs and renal allograft survival. The results showed that high expression levels of EZH2, CDKN1A, EGR1, ATF3, and CD44 were all associated with poor prognosis, while low expression of PPARA was associated with poor prognosis (**Figure 5C**). These experimental results indicate that EZH2, ATF3, EGR1, CDKN1A, and CD44 can be upregulated early after IR and their sustained high expression levels are associated with poor prognosis, while PPARA initially decreases in expression but can gradually recover, and its increased expression levels are associated with good prognosis.

### Immune cell infiltration and correlation analysis of FRHGs and immune cells

**Figure 6:**
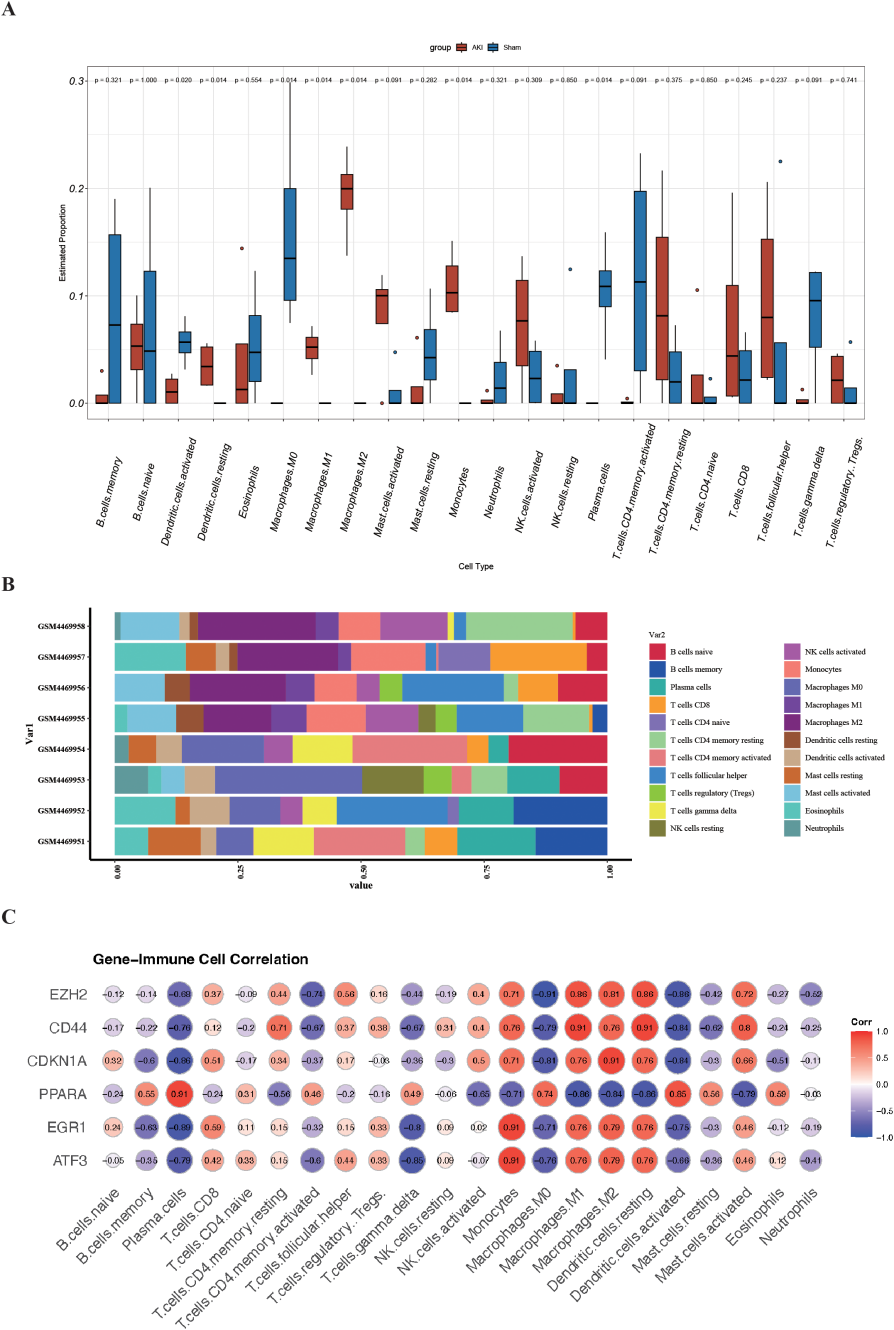
Analysis of immune cell infiltration and correlation with FRHGs in renal IRI. **(A)** Box plot shows the expression of 22 immune cells in renal IRI. **(B)** stacked plots of the expression of 22 immune cells in each sample. **(C)** correlation analysis between FRHGs and immune cells.

In the GO and KEGG enrichment analysis of the DEFRGs, immune cells appear to play a crucial role in the process of renal IRI. To further confirm the role of immune cells, we conducted immune infiltration analysis and found significant differences (P<0.05) in seven immune cells between the IRI and Sham groups, namely: Dendritic cells activated, Dendritic cells resting, Macrophages M0, Macrophages M1, Macrophages M2, Monocytes, and Plasma. cells (**Figures 6A, B**). To better understand the role of FRHGs in immune infiltration, we used Spearman correlation analysis to determine their association with immune cell infiltration. The correlation analysis showed that EZH2, CDKN1A, PPARA, EGR1, ATF3, and CD44 were strongly correlated with all seven immune cells. Among them, EZH2 showed the strongest negative correlation with Macrophages.M0; CD44 showed strong positive correlation with Macrophages M1 and Dendritic cells resting; CDKN1A showed strong positive correlation with Macrophages M2; PPARA showed strong positive correlation with Plasma cells; and EGR1 and ATF3 showed strong positive correlation with Monocytes (**Figure 6C**).

### Identification of potential drugs for FRHGs interactions

**Figure 7:**
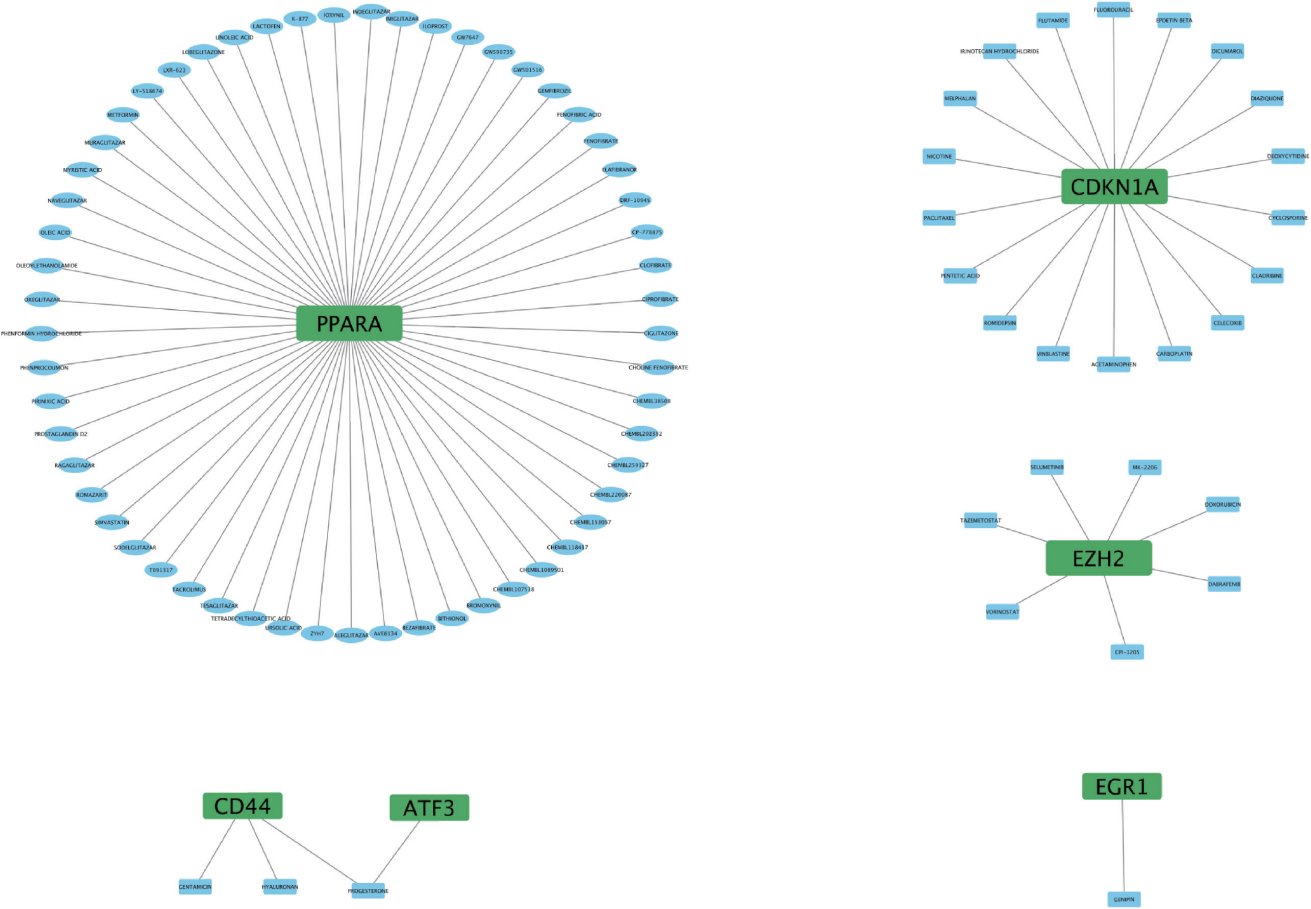
Drug interaction network with EZH2, CDKN1A, PPARA, EGR1, ATF3, and CD44 genes.

To further understand the interaction between FRHGs and drugs, we predicted drugs or molecular compounds that may interact with FRHGs based on the DGIDB database. A total of 87 drugs or molecular compounds that may have regulatory relationships with FRHGs were screened, of which the largest number of drugs interacting with PPARA was identified, with a total of 57 related drugs (**Figure 7**).

### Validation of FRHGs protein expression by proteomics and Western blot

**Figure 8:**
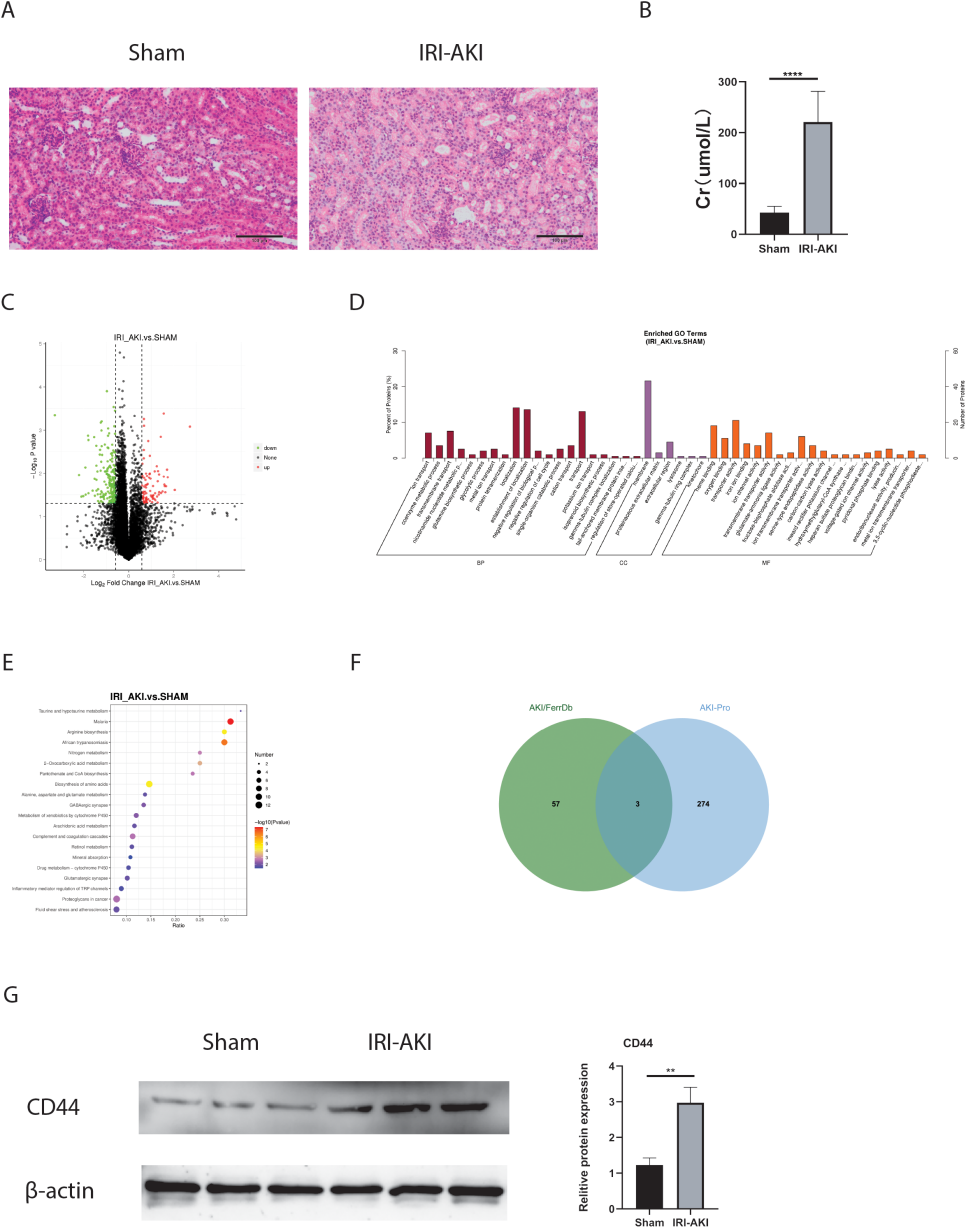
FRHGs protein expression of IRI-AKI. **(A)** The representative HE staining image of IRI-AKI. **(B)** The level of creatinine. **(C)** Volcano plot of differentially expressed proteins in IRI-AKI. **(D)** GO analysis of DEPs. **(E)** KEGG analysis of DEPs. **(F)** Intersection of FRHGs and DEPs. **(G)** CD44 proteins Level detected by Western blot. **** represents P<0.0001, ** represents P<0.01.

As we all know that protein is the primary executor of gene function. In this study, Proteomics and Western blot were further used to confirm the protein expression of FRHGs. Hematoxylin and eosin (HE) staining and creatinine determination confirmed successful modeling (**Figures 8A, B**). Differentially expressed proteins (DEPs) between IRI-AKI and Sham groups were analyzed in R software. The results showed 88 proteins exhibiting an upregulation trend, and 198 proteins exhibiting a downregulation trend (**Figure 8C**). GO analysis showed that “ion transport [BP],” “membrane [CC],” “ion channel activity [MF],” and “iron ion binding [MF]” were the most significantly enriched biological processes (**Figure 8D**). In KEGG, “Arachidonic acid metabolism” was the primarily enriched pathway (**Figure 8E**). Among these, the iron ion binding pathway was the commonly enriched biological process between DEFRGs and DEPs. By overlapping DEFRGs with differentially expressed proteins, we identified three intersecting proteins, among which CD44 was the only FRGH with elevated expression at both the gene and protein levels (**Figure 8F**). Subsequently, we further validated the protein expression levels of CD44 using Western blot (**Figure 8G**).

### Blockade of CD44 mitigates renal tissue injury following ischemia-reperfusion through inhibition of ferroptosis

**Figure 9:**
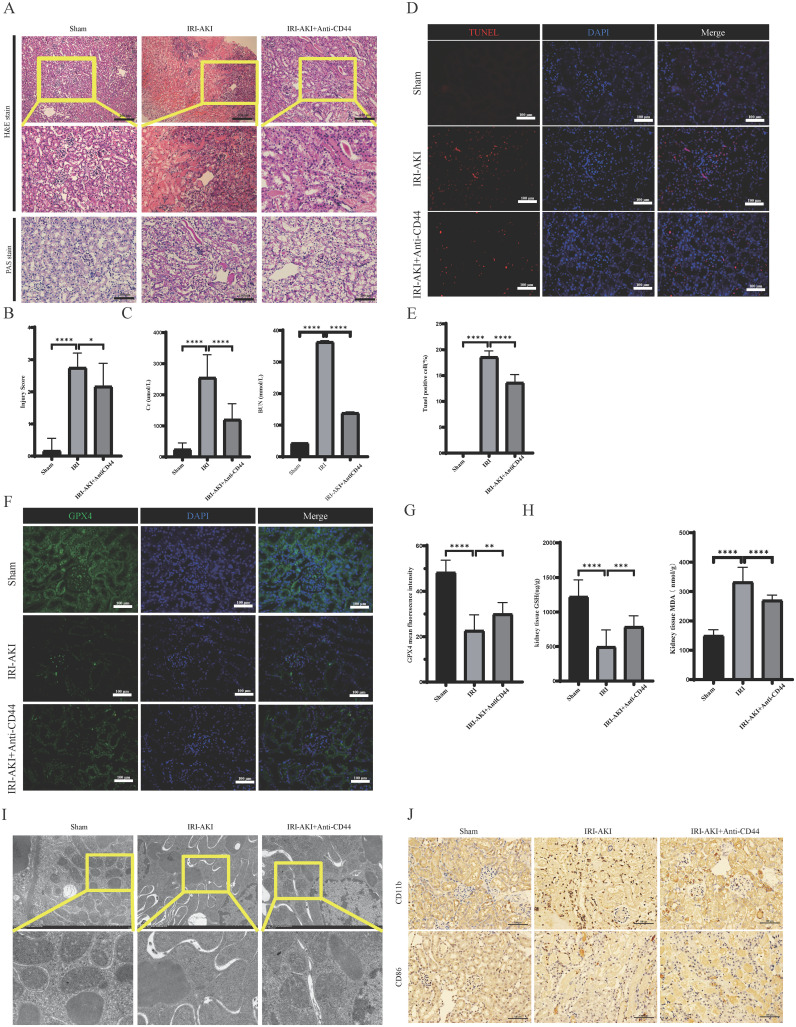
Blockade of CD44 inhibits the accumulation of macrophages and alleviates ferroptosis during the development of renal IR. **(A)** Representative image of HE and Pas staining. **(B)** Damage score of renal tubular injury; **(C)** Renal function detection; **(D)** The representative image of TUNEL staining; **(E)** The percentage of Tunel positive cell. **(F)** The representative image of immunofluorescence staining of GPX4; **(G)** Mean fluorescence intensity of GPX4. **(H)** GSH and MDA level in kidney tissue. **(I)** The representative image of transmission electron microscopy. **(J)** Macrophages infiltrated into kidney. **** represents P<0.0001, *** represents P<0.001, ** represents P<0.01, * represents P<0.05.

To further elucidate the critical role of CD44 in IRI-AKI, we established a mouse model of IRI-AKI and administered CD44-blocking antibodies (Anti-CD44). Compared to the Sham group, the IRI-AKI group exhibited tissue damage characterized by tubular necrosis, epithelial cell shedding, and inflammatory cell infiltration (**Figure 9A**). Renal function assessments revealed that Scr and BUN levels were increased rapidly; however, pre-treatment with Anti-CD44 significantly mitigated histological injury and functional impairment in the kidneys (**Figures 9A–C**). We also investigated the role of CD44 in ferroptosis. TUNEL staining indicated high death rate in renal tissues following IRI, accompanied by significant declines in ferroptosis-related indicators such as glutathione (GSH) and GPX4, and a marked increase in MDA levels (**Figures 9D–H**). Transmission electron microscopy demonstrated diminished, degenerated, or absent mitochondrial cristae in the IRI-AKI group (**Figure 9I**). Pre-treatment with CD44 monoclonal antibodies effectively reduced ferroptosis, evidenced by a decrease in TUNEL positive cells, an increase in GSH levels, elevated GPX4 expression, and a reduction in MDA, as well as lessened mitochondrial structural damage (**Figures 9D–I**). These findings suggest that CD44 plays a protective role in inhibiting ferroptosis during renal IRI. Furthermore, immunohistochemical analyses indicated a significant increase in M1 macrophage accumulation during IRI, which was effectively reduced by Anti-CD44 treatment(**Figure 9J**). The relationship between reduced M1 macrophage accumulation and the inhibition of ferroptosis warrants further investigation.

## Discussion

Acute kidney injury is a clinical syndrome characterized by a rapid decline in kidney function, such as glomerular filtration rate and endogenous creatinine clearance rate, within a short period of time. It has a high incidence and mortality rate. Although renal replacement therapy is becoming increasingly sophisticated, progress in identifying targets or treatment strategies to improve AKI outcomes has been slow. Ferroptosis is a unique form of cell death driven by iron-dependent lipid peroxidation, characterized by iron overload, accumulation of reactive oxygen species, and lipid peroxidation (2). Recent studies have shown that ferroptosis may represent a potential therapeutic target for kidney diseases, including AKI (9, 10). This study aims to identify important ferroptosis-related genes in IRI-AKI through bioinformatics analysis.

Iron accumulation and lipid peroxidation are two key signals that trigger membrane oxidative damage in the ferroptosis process (11). Iron accumulation may affect ferroptosis in three ways: the involvement of iron in the catalytic activity of metabolic enzymes LOXs and POR required for phospholipid peroxidation; iron is essential for enzymes involved in ROS generation; non-enzymatic, iron-dependent Fenton chain reactions may be necessary for ferroptosis (2). Additionally, polyunsaturated fatty acids (PUFAs), especially arachidonic acid and adrenic acid, are most susceptible to lipid peroxidation, leading to the destruction of the lipid bilayer and affecting membrane function (12, 13). In our study, we performed GO and KEGG enrichment analyses and found that DEFRGs were mainly enriched in “response to lipid (BP)”, “iron ion binding (MF)”, and “ferroptosis”, which is interesting since lipid peroxidation is an important feature of ferroptosis. Proteins are one of the main carriers of biological functions and substance transport. Therefore, we used proteomics to explore the major changes in differential proteins in biological processes and pathways in IRI-AKI. The results showed that “ion transport (BP)”, “membrane (CC)”, “ion channel activity (MF)”, and “iron ion binding (MF)” were the most significantly enriched biological processes. In the KEGG pathway analysis, “Arachidonic acid metabolism” was the major enriched pathway. Among them, “iron ion binding” was a common enriched biological process for both DEFRGs and DEPs. Animal studies have shown that in IRI-AKI, STING promotes ferroptosis through NCOA4-dependent ferritinophagy (14), and inhibiting ferroptosis can alleviate drug or sepsis-induced kidney injury (15, 16). In our study, we elucidated that ferroptosis play a significant role in IRI-AKI, primarily manifested as an increase in MDA levels, a decrease in GSH levels, the reduction or disappearance of mitochondrial cristae, and a decrease in the expression of GPX4. In general, through the GO and KEGG analysis of transcriptomics and proteomics, we found multiple pathways or biological processes enriched in renal IRI related to iron transport and lipid metabolism. Through animal models, we have confirmed the occurrence of ferroptosis in IRI-AKI. Therefore, we believe that ferroptosis plays a crucial role in the process of IRI-AKI, and its mechanism may be related to iron accumulation and arachidonic acid metabolism.

Through KEGG enrichment analysis, we observed that DEFRGs were also enriched in “Toll-like receptor signaling pathway” and “NOD-like receptor signaling pathway”. This suggests that ferroptosis-related genes may be involved in immune regulation and cannot be ignored in the process of renal IRI. Therefore, we performed immune infiltration analysis to further elucidate the relationship between FRGHs and immune cells. Our study suggests that PPARA is strongly positively correlated with Plasma. cells, and ATF3 and EGR1 are strongly positively correlated with Monocytes, while CD44 is strongly positively correlated with M1 Macrophages. EZH2 shows a negative correlation with M0 macrophages and a strong positive correlation with M2 macrophages. This suggests that CD44 and EZH2 may play important roles in macrophage polarization. Enhancer of zeste homolog 2 (EZH2) belongs to the family of polycomb group genes (PcGs), a crucial group of epigenetic regulators that are responsible for suppressing transcription (17). Research shows that EZH2 works as a master regulator of cell cycle progression (18), autophagy, and apoptosis (19), promotes DNA damage repair and inhibits cellular senescence (20) and plays an important role in cell lineage determination and relative signaling pathways (20). Reportedly, Targeting EZH2 protects against acute kidney injury via Raf-1/ERK1/2 pathway (21). What’s more interesting, Targeted inhibition of EZH2 may improve renal fibrosis after acute kidney injury by counteracting partial EMT and blockade of M2 macrophage polarization (22). CD44, a kind of cell-surface glycoprotein, plays a role in angiogenesis, cytoskeleton rearrangement, tumor proliferation, cell adhesion, and migration (23).In recent years, studies have shown that a CD44-targeted hyaluronic acid-curcumin prodrug protects renal tubular epithelial cell survival from oxidative stress damage (24).Interestingly, CD44 contributed to the recruitment of monocyte/macrophages to the kidney following IRI (25). In our study, CD44 was the only FRGHs that was highly expressed at both the gene and protein levels. Further animal experiments confirmed that blocking CD44 inhibits ferroptosis and the accumulation of M1 macrophage, exerting a protective effect during IRI-AKI. However, the connection between M1 macrophages and ferroptosis requires further investigation.

As one of the main pathophysiological processes in kidney transplantation, IRI is closely related to the clinical prognosis of patients (26). Therefore, we investigated the impact of FRHGs on long-term graft survival. Survival analysis results showed that high expression levels of EZH2, CDKN1A, EGR1, ATF3, and CD44 were all associated with poor prognosis, while high expression of PPARA was associated with good prognosis. Among them, Early growth response 1 (EGR1) is an “immediate early” transcription factor that plays an important role in the migration of breast tumors (27). In normal adult kidneys, EGR1 expression is almost undetectable (28). EGR1 begins to rise immediately after IR and begins to decline slowly after peaking (29). ATF3 is a member of the ATF/CREB subfamily of the basic leucine zipper (BZIP) family. Previous studies have shown that ATF3 can be detected in urine within 2-24 hours in a rat model of AKI induced by ischemia reperfusion injury and is considered a novel diagnostic biomarker for AKI (30–32). PPARA has also been shown to have a protective role in IRI-AKI (33). Combined with our findings, these prognostic genes may be deeply involved in the progression of renal IRI, thereby having beneficial or harmful effects on long-term outcomes in post-transplant patients, and may serve as effective therapeutic targets. In fact, some studies have provided a theoretical basis for EGR1, ATF3, and PPARA as therapeutic targets (29, 34), and the clinical applicability needs to be further studied.

Finally, we predict that 87 drugs or molecular compounds may be involved in the regulation of Hub gene, which could be a potential anti-IRI-AKI drug. Many studies have already demonstrated the impact of drugs or molecular compounds on renal IRI. For example, Cyclosporine and Fenofibrate (PPARA) attenuate IRI-AKI in rat ischemia reperfusion model (35, 36). Dabrafenib alleviates kidney IRI by inhibiting cell death and suppressing inflammatory responses (37). Selective PPARA agonist GEMFIBROZIL may exert a protective role in IRI-AKI by reducing podocyte apoptosis (38); Celecoxib beneficially affects the outcome of renal IRI by reducing oxidative stress through decreasing COX-2 expression (39). These drugs or molecular compounds could be potential drugs for future treatment of IRI-AKI.

In conclusion, through bioinformatics analysis, proteomics analysis, and animal experiments, we identified six FRHGs during renal IRI: EZH2, CDKN1A, PPARA, EGR1, ATF3, and CD44. Among them, only CD44 was overexpressed at both the gene and protein levels. Anti-CD44 exerts a protective effect by inhibiting ferroptosis and the accumulation of M1 macrophages during renal IRI. However, the connection between M1 macrophages and ferroptosis requires further investigation.

The limitations of this study mainly include the following: the transcriptional data were obtained from different datasets in the GEO database, where different sequencing platforms and batch effects may affect the reliability of experimental results. Additionally, the proteomic sequencing results were self-generated data, thus potential batch effects could exist. Moreover, the sequencing data in this study included samples from both mice and humans, and insufficient consideration of inter-species differences limited the further clinical application of the study results, requiring more experiments for further validation.

## Conclusions

In summary, we conducted comprehensive bioinformatics analyses of the IRI-AKI model and identified 6 ferroptosis-related Hub genes. We utilized proteomics to further validate the expression of ferroptosis-related proteins and discovered multiple pathways and biological processes related to ferroptosis were enriched in IRI-AKI. We found that ferroptosis-related hub genes not only exhibited significant correlation with immune cells but also directly influenced the survival of transplanted kidneys in the human population. Among six FRHGs, only CD44 was overexpressed at both the gene and protein levels. Anti-CD44 exerts a protective effect by inhibiting ferroptosis and the accumulation of M1 macrophages during renal IRI. The study also predicted 87 drugs that may act on IRI-AKI, some of which have been confirmed effective. These findings provide potential therapeutic targets and a deeper understanding of the mechanisms of IRI-AKI and offer novel perspectives on the diagnosis and treatment of IRI-AKI.

## Data Availability

The original contributions presented in the study are included in the article/supplementary materials. Further inquiries can be directed to the corresponding authors.
